# Supplementary figures and images for: Enhanced monocyte migratory activity in the pathogenesis of structural remodeling in atrial fibrillation
Source: PLoS One. 2020 Oct 13;15(10):e0240540. doi: 10.1371/journal.pone.0240540 (PMC7553285; doi:10.1371/journal.pone.0240540)

**
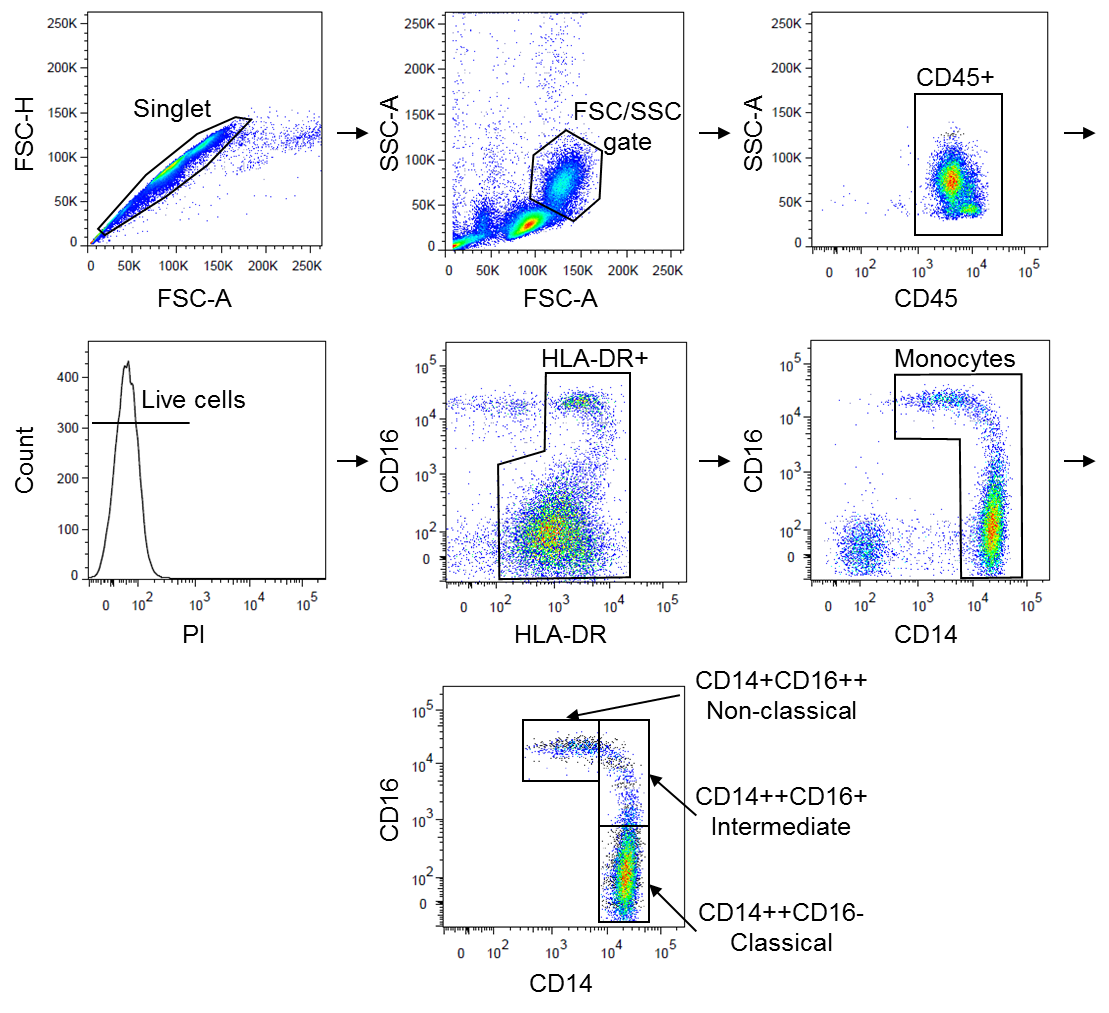
**

Supplement: S1 Fig — Doublets were excluded with forward scatter (FSC)-A versus FSC-H plots, then gated on cells including monocytes based on FSC / side scatter (SSC) plots. CD45 positive, propidium iodide (PI) negative and HLA-DR positive population was further selected. After gating-out CD14-CD16- cells, monocytes were classified into three subsets. (DOCX) [file pone.0240540.s001.docx]

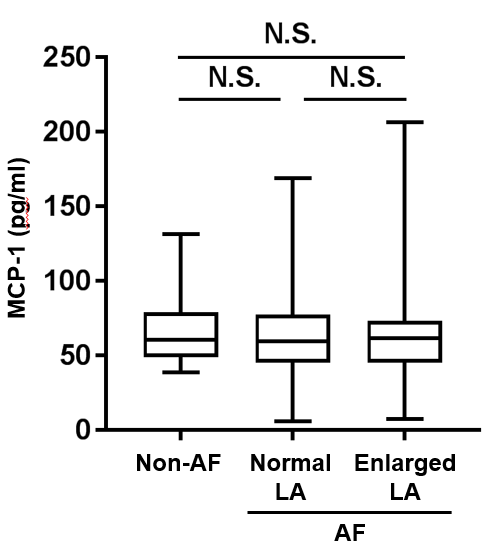

Supplement: S2 Fig — Serum MCP-1 levels were determined by human MCP-1 DuoSet ELISA (R&D, Minneapolis, MN). N.S.: not significant (non-AF: n = 21, normal LA: n = 80, enlarged LA: n = 76, Tukey’s test). (DOCX) [file pone.0240540.s002.docx]

Fig 3 CCR2

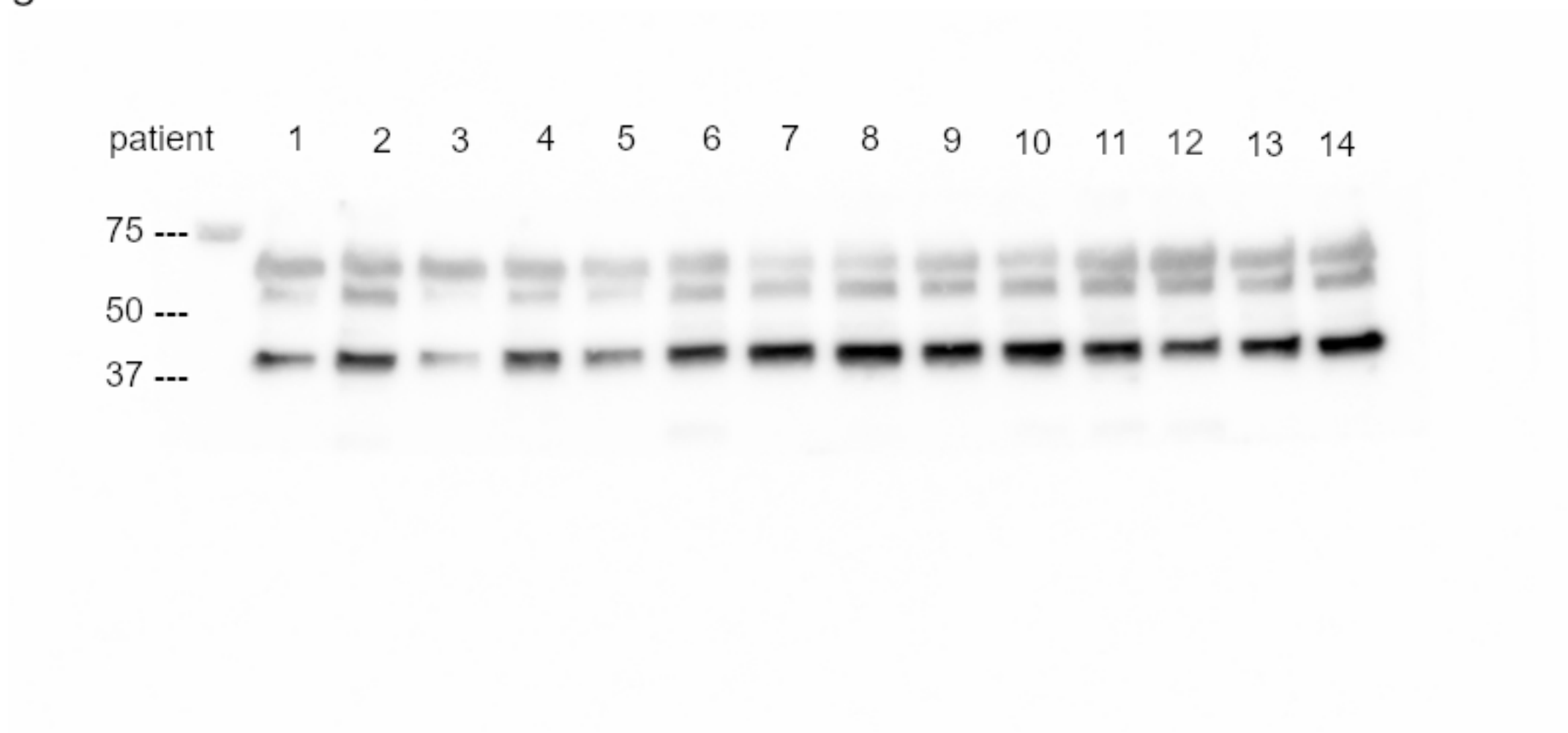

Fig 3 CypB

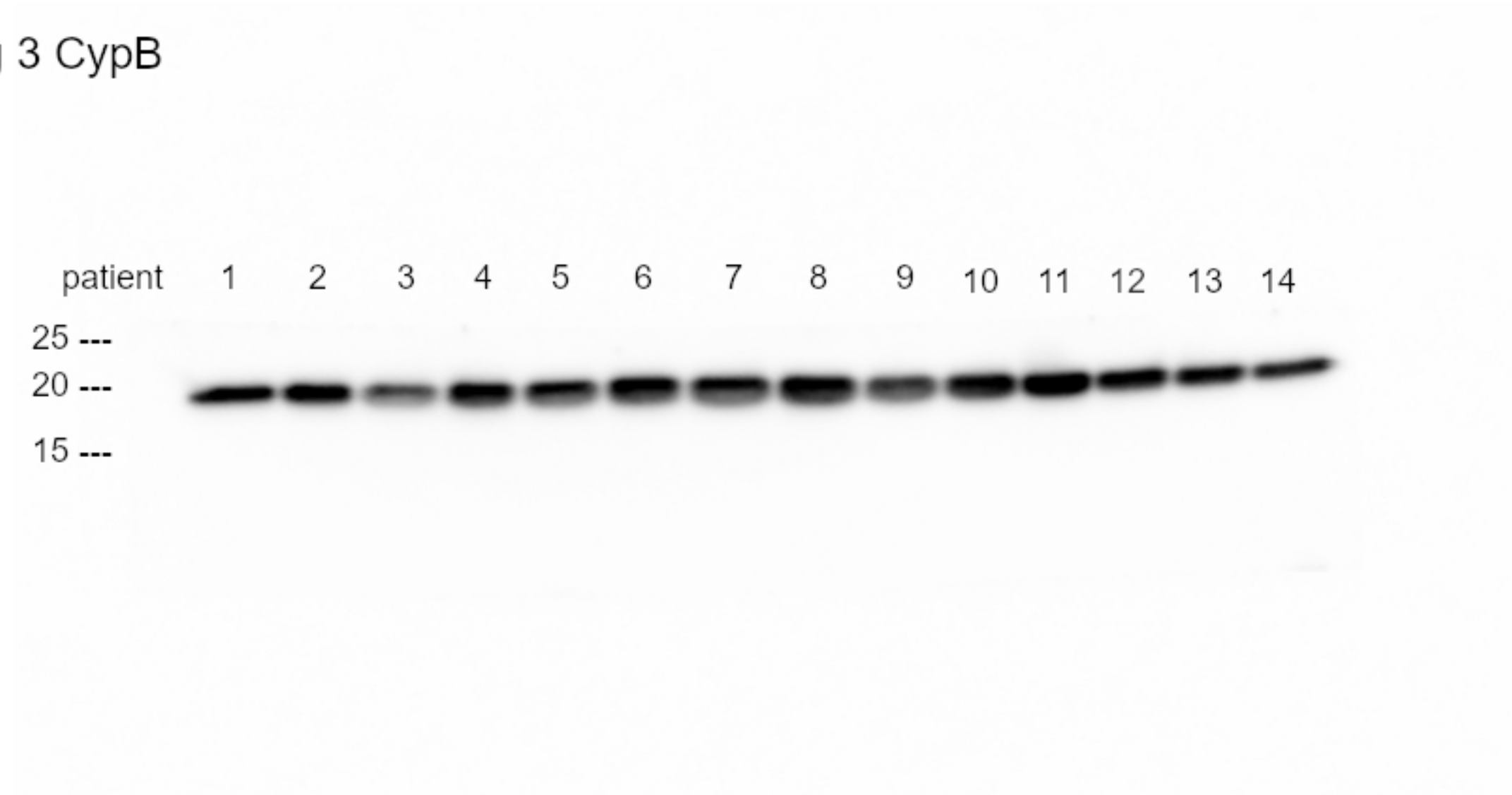

Supplement: S1 Raw images — (PDF) [file pone.0240540.s003.pdf]
